# Case report: Dihydropyridine receptor (*CACNA1S*) congenital myopathy, a novel phenotype with early onset periodic paralysis

**DOI:** 10.3389/fneur.2024.1359479

**Published:** 2024-02-15

**Authors:** Samah K. Aburahma, Liqa A. Rousan, Mohammad Shboul, Fabio Biella, Sabrina Lucchiari, Giacomo Pietro Comi, Giovanni Meola, Serena Pagliarani

**Affiliations:** ^1^Department of Pediatrics, Jordan University of Science and Technology, Irbid, Jordan; ^2^Department of Radiology, Jordan University of Science and Technology, Irbid, Jordan; ^3^Department of Laboratory Sciences, Jordan University of Science and Technology, Irbid, Jordan; ^4^Neurology Unit, Foundation IRCCS Ca' Granda Ospedale Maggiore Policlinico, Milan, Italy; ^5^Neuroscience Section, Dino Ferrari Centre, Department of Pathophysiology and Transplantation (DEPT), University of Milan, Milan, Italy; ^6^Department of Neurorehabilitation Sciences, Casa di Cura Igea, Milan, Italy; ^7^Department of Biomedical Sciences for Health, University of Milan, Milan, Italy

**Keywords:** congenital myopathy, episodic weakness, *CACNA1S*, Ca_v_1.1, DHPR, splice minigene assay, novel phenotype, periodic paralysis

## Abstract

**Introduction:**

*CACNA1S* related congenital myopathy is an emerging recently described entity. In this report we describe 2 sisters with mutations in the *CACNA1S* gene and the novel phenotype of congenital myopathy and infantile onset episodic weakness.

**Clinical description:**

Both sisters had neonatal onset hypotonia, muscle weakness, and delayed walking. Episodic weakness started in infancy and continued thereafter, provoked mostly by cold exposure. Muscle imaging revealed fat replacement of gluteus maximus muscles. Next generation sequencing found the missense p.Cys944Tyr variant and the novel splicing variant c.3526-2A>G in *CACNA1S*. Minigene assay revealed the splicing variant caused skipping of exon 28 from the transcript, potentially affecting protein folding and/or voltage dependent activation.

**Conclusion:**

This novel phenotype supports the notion that there are age related differences in the clinical expression of *CACNA1S* gene mutations. This expands our understanding of mutations located in regions of the *CACNA1S* outside the highly conserved S4 segment, where most mutations thus far have been identified.

## 1 Introduction

In skeletal muscle, action potential propagation results in muscle contraction, a process mediated by calcium ions and known as excitation-contraction coupling (ECC) (1). Specialized proteins take part in ECC, including the dihydropyridine receptor (DHPR), a voltage gated calcium channel located on T-tubule membranes and RyR1, located on the sarcoplasmic reticulum (SR). T-tubules tightly associate with terminal cisternae of the SR; the close association between one T-tubule and two terminal cisternae form the triad and the interaction between DHPR and RyR1 upon depolarization activates opening of RyR1 (1).

The α1 subunit of the DHPR contains the basic functional elements of the L-type Ca^2+^ channel 1.1 (Ca_v_1.1) and is encoded by the *CACNA1S* gene. The α1 subunit is formed by four homologous domains (DI-DIV) and each domain contains six transmembrane segments (S1–S6) (2). In each domain, segments S1–S4 form the voltage-sensing domain, whereas S5 and S6 from all four domains form the pore with its activation gate. The activation of DHPR triggers a rapid elevation of cytosolic Ca^2+^ thus coupling membrane excitation to muscle cell contraction (3).

The classical adult skeletal muscle L-type calcium currents are small, activate slowly and open only at 30 mV more positive membrane potentials than EC coupling (4). In embryonic tissues, the major isoform expressed lacks exon 29 (Ca_v_1.1e) and exhibits high current amplitude, fast activation kinetics and normal voltage sensitivity compared to the adult isoform (5). After birth the embryonic isoform is rapidly substituted by the adult isoform containing exon 29 (Ca_v_1.1a) (6).

Mutations in the *CACNA1S* gene have been previously associated with malignant hyperthermia susceptibility (7) and hypokalemic periodic paralysis (HypoPP) (8, 9). A recent report correlated *CACNA1S* mutations to both recessive and dominant forms of congenital myopathy (10). Only few patients have been described so far having recessive *CACNA1S*-related congenital myopathy.

Here, we describe 2 sisters from a consanguineous family who presented with the novel phenotype of congenital myopathy and early onset episodic weakness. Two likely pathogenic variants in the *CACNA1S* gene were identified in both sisters.

## 2 Case report

### 2.1 Clinical description

The two probands are sisters of consanguineous healthy parents (second degree cousins). In addition to the two probands, there are two healthy sisters. The parents gave written informed consent for genetic and research studies in accordance with guidelines provided by the Institutional Review Board of North Jordan, King Abdullah University Hospital, Irbid, Jordan.

Proband 1 was a term newborn, via normal vaginal delivery. At birth she had significant head lag and hypotonia. She continued to have poor head control requiring support, poor limb movements, and poor weight bearing. There were no feeding or breathing concerns. Walking was delayed till after 2 years of age. Cardiac evaluation was normal. Around seven to 10 months of age, episodes of weakness, and flaccidity manifesting as “falling over” while seated started, particularly with cold exposure. Episodes were around 10 min duration, and continued to occur once or twice a month, more so in the winter period and with cold ambient temperature. With advancing age, she continued to have episodic loss of ambulation, particularly with cold exposure, but also with physical exertion. Her neurological exam at 12 years of age was notable for weakness involving neck flexion and proximal limb muscles (shoulder abduction, forearm flexion, hip flexion) MRC grade 4/5. Deep tendon reflexes were normal. Cranial nerve examination revealed mild facial weakness, without ophthalmoplegia, ptosis, or bulbar weakness.

The younger sister of proband 1 (II.4) presented at 4 years of age. She was the product of a full-term uncomplicated pregnancy via normal vaginal delivery. She, also, was noted to have significant hypotonia shortly after birth, with poor head control, and very little antigravity movements. Walking was delayed till after 2 years of age. There were no concerns regarding feeding and breathing. Around 7 months of age, she started having episodes of flaccidity and falling over if seated, particularly with cold exposure or cold ambient weather. These episodes lasted around 10 min. Her neurological examination at 4 years of age was notable for a weak myopathic appearing face, but otherwise no clear weakness or fatigability were observed. There was no clinical myotonia. Deep tendon reflexes were normal. For both probands, there was poor response to treatment with either acetazolamide or a low carbohydrate diet for prevention of episodic weakness. A timeline of the clinical course is presented in Table 1.

**Table 1:** Timeline of clinical events for proband 1 and proband 2.

	**Year (age of patient)**	**Clinical event**	**Diagnostic procedure**
Proband 1	Newborn period	Floppy, poor head control, muscle weakness and poor limb movements	
	7–10 months of age	Episodic “falling over,” 10 min duration of weakness and inability to sit up, aggravated by cold exposure	
	2 years of age	Noted delayed walking	Normal CPK
	6 years of age	Continued episodic weakness, exam notable for weak myopathic face	Nerve conduction studies: normal motor and sensory responses Electromyographic myotonic discharges
	12 years of age	Mild proximal muscle weakness Continued episodic weakness	Nerve conduction studies: normal motor and sensory responses Long exercise test: no drop of baseline CMAP over time or in relation to exercise Muscle MRI: symmetrical tigroid pattern fat replacement, muscle atrophy NGS: two variants in *CACNA1S* gene
Proband 2	Newborn period	Floppy, poor head control, muscle weakness, and poor limb movements	
	7–10 months of age	Episodic “falling over,” 10 min duration of weakness and inability to sit up, aggravated by cold exposure	
	2 years of age	Noted delayed walking	Normal CPK
	4 years of age	Continued episodic weakness, exam notable for weak myopathic face	Nerve conduction studies: normal motor and sensory responses Electromyographic myotonic discharges NGS: two variants in *CACNA1S* gene

### 2.2 Diagnostic assessment

Proband 1: Laboratory evaluation revealed normal creatine kinase (CK). Electrophysiological evaluation revealed normal motor and sensory nerve conduction studies. Long exercise testing for median nerve/abductor pollicis brevis muscle and ulnar nerve/abductor digiti minimi muscle was normal. Limb cooling and cold provocation test did lead to clinical weakness, and significant drop in motor response.

A previous EMG of right tibialis anterior, extensor indices, and deltoid muscles performed when she was 6 years old revealed myotonic discharges in tibialis anterior and extensor indices muscles.

Repeat EMG of right tibialis anterior, vastus lateralis, first dorsal interosseus, extensor indices and biceps muscles at age 12 was normal.

**Figure 1:**
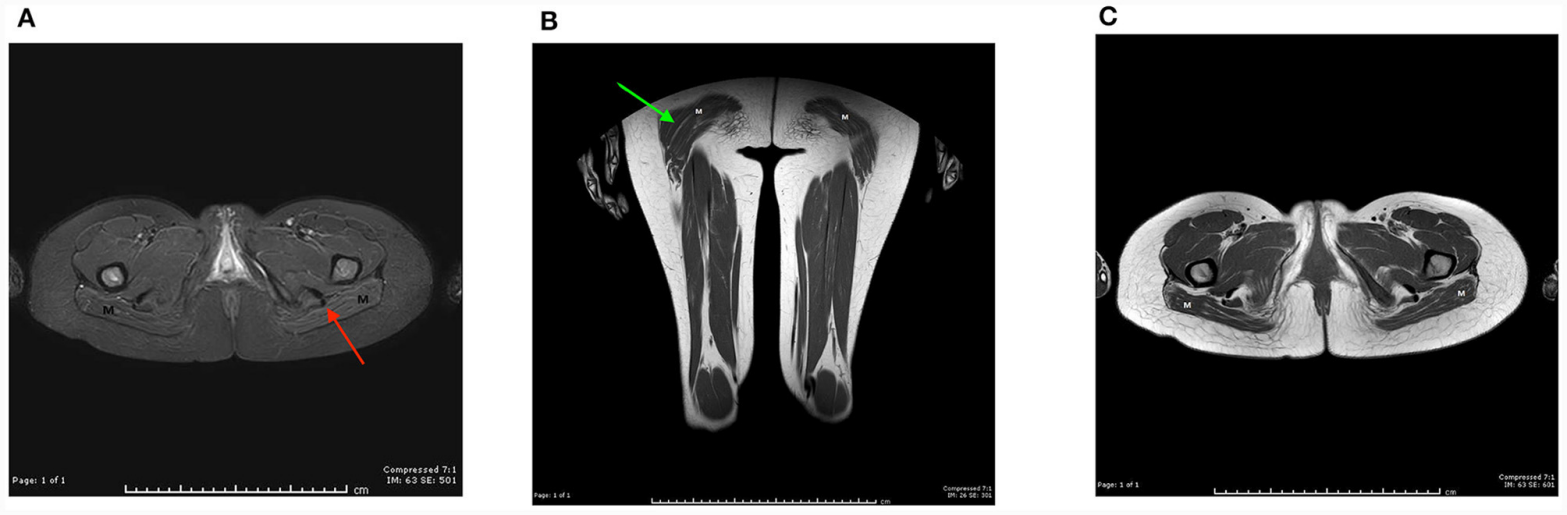
MRI of the pelvis, thighs, upper limbs, and neck was performed on proband 1 using a 1.5T scanner (Toshiba, USA). Axial and coronal T1 and STIR sequences were obtained using a sense neuro vascular coil for the neck, and a multi coil for the rest. Axial T1 image at the level of the pelvic floor **(A)**, and coronal T1 image of the thigh, **(B)** show symmetrical fat replacement of both gluteus maximus muscles (M), red and green arrows, respectively. The pelvic and thigh muscles bilaterally appear uninvolved. **(C)** Axial STIR images show suppression of the intramuscular fat (M).

For proband 1, around 6 weeks after last clinical evaluation detailed above, MRI of the pelvis, thighs, upper limbs, and neck was performed using a 1.5T scanner (Toshiba, USA). Axial and coronal T1 and STIR sequences were obtained and showed atrophy and symmetrical fat replacement of the gluteus maximus muscles in a tigroid pattern, of an estimated 30–60% replacement (Figure 1). The rest of the examined muscles were uninvolved.

Proband 2: CK was normal. Limited neurophysiological evaluation revealed normal motor nerve conduction studies, with evidence of myotonic discharges in the tibialis anterior muscle.

Serum potassium status during the episodes of weakness is unknown; this proved to be a challenging aspect of their care due to the short duration of episodes, coupled with their rural residence and distance from medical facilities.

### 2.3 Genetic evaluation

Genetic evaluation was not available in Jordan till proband 1 was 12 years of age, with the availability of next generation sequencing (NGS) utilizing sputum samples which made it feasible to ship overseas. Sanger sequencing and minigene assay was possible through the cooperation with Biochemistry and Genetic lab of the Neurology Unit of IRCCS Ospedale Maggiore Policlinico (Milan, Italy).

**Figure 2:**
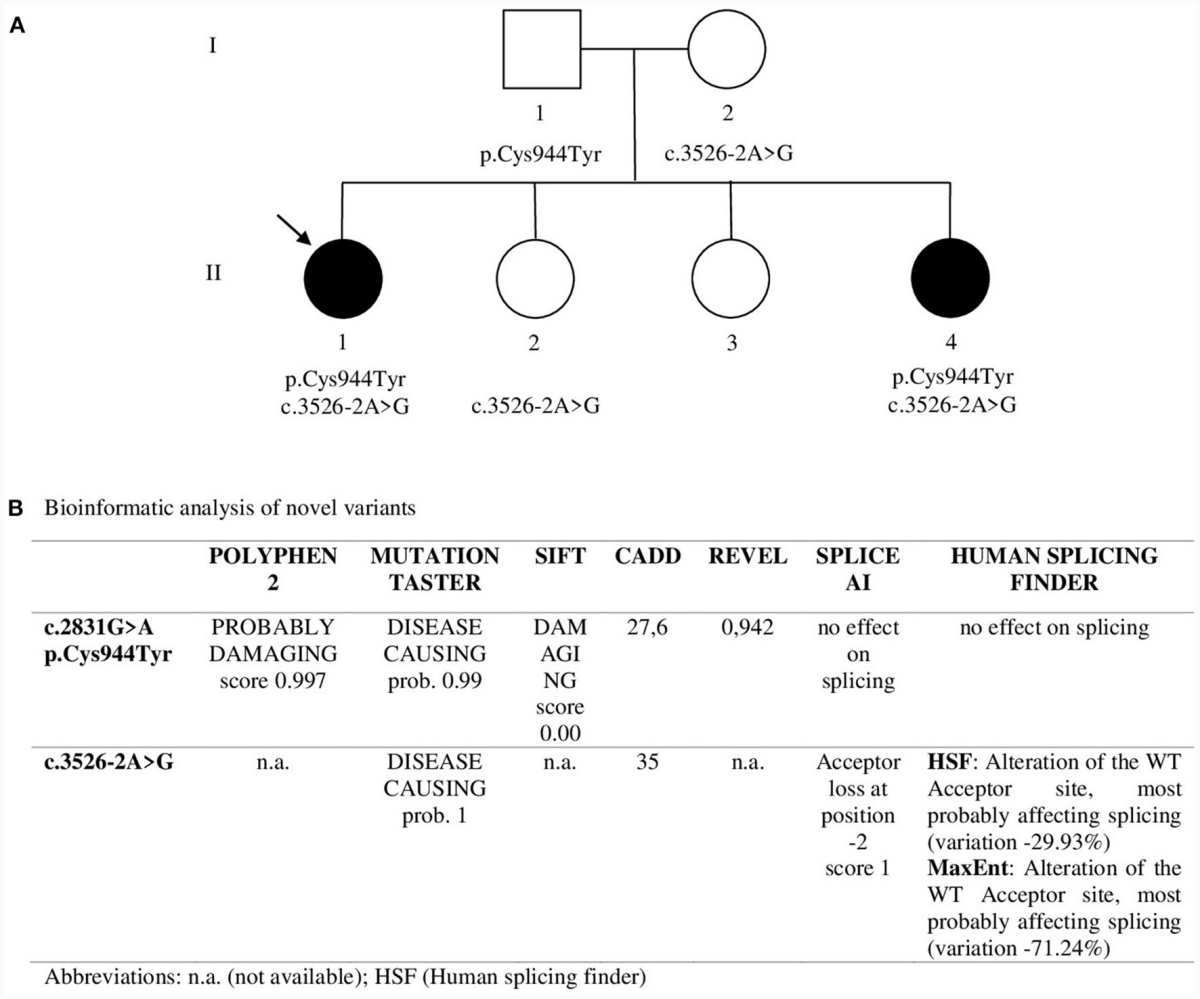
**(A)** Pedigree. Sanger sequencing was used to study segregation in family members. Asymptomatic sister II.2 showed only c.3526-2A>G in heterozygosity while asymptomatic sister II.3 showed neither of the two variants. The arrow indicates Proband 1. **(B)** Bioinformatic analysis of novel variants.

NGS analysis was performed by Invitae using the Invitae Comprehensive Neuromuscular Disorders Panel customized to include 109 genes (the complete gene list is available at https://www.invitae.com/us/providers/test-catalog/test-03280). In proband 1 two variants in *CACNA1S* were revealed: c.2831G>A (p.Cys944Tyr) in exon 22 and c.3526-2A>G at the acceptor splice site of intron 27. Sanger sequencing confirmed the segregation of both variants in the two affected sisters: c.3526-2A>G was inherited from the unaffected mother and c.2831G>A from the unaffected father, thus revealing a recessive pattern of inheritance (Figure 2A). p.Cys944Tyr replaces a highly conserved cysteine residue with tyrosine at codon 944 in the DIII-S5 of Ca_v_1.1. Cys944Tyr is described in GnomAD with allele frequency 1.59 × 10^−6^; Clinvar and dbSNP assigned ID codes 1017723 and rs1661217137, respectively. *In silico* analysis predicted a deleterious impact of this variant on channel function (Figure 2B). funNCion, an online tool which predicts functional effects of missense variants in voltage-gated sodium and calcium channels, predicted p.Cys944Tyr to be pathogenic with a probability of 0.95 and to be loss-of-function with a probability of 0.69. ACMG rules classified p.Cys944Tyr as likely pathogenic (PM2_supporting, PM3, PP1, PP3_strong). c.3526-2A>G lay on the acceptor splice site of intron 27 and is described in GnomAD with allele frequency 1.20 × 10^−6^. Clinvar and dbSNP assigned ID codes 209137 and rs797045031, respectively. *In silico* prediction programs suggested a damaging effect causing a possible alteration of pre-mRNA splicing (Figure 2B). ACMG rules classified this variant as likely pathogenic (PS3, PM2_supporting, PM4, PP1, PP3).

**Figure 3:**
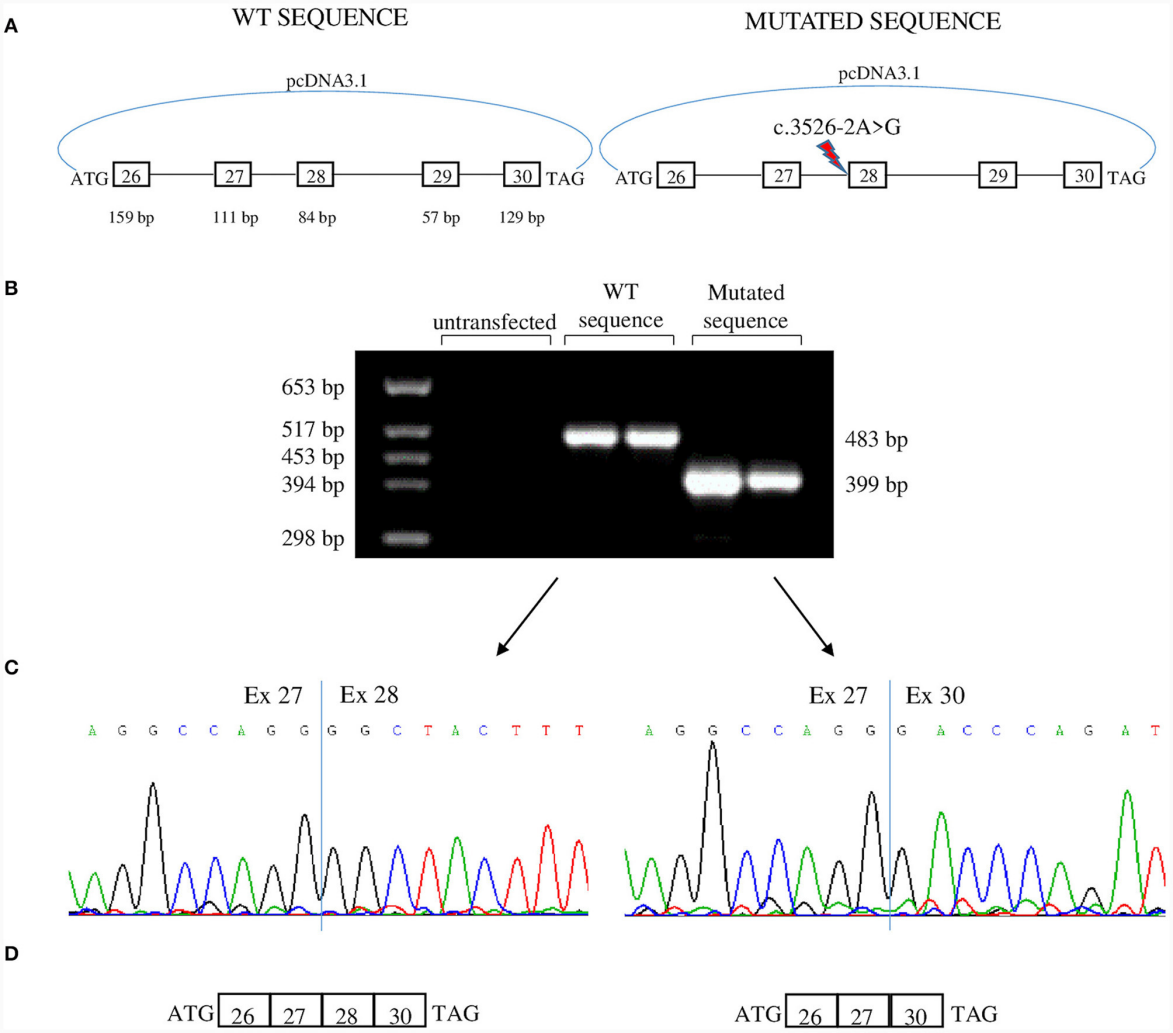
Minigene assay. **(A)** Both c.3526-2A>G allele and WT allele from one of the patients were cloned in pcDNA3.1 plasmid and were transfected in tsA human epithelial kidney (HEK) cells. The genomic region cloned encompassed exons 26–30. **(B)** Agarose gel showing RT-PCR products derived from minigenes. The product from c.3526-2A>G allele is smaller than that of the WT allele. **(C)** Sequence electropherograms showing the effect of c.3526-2A>G in the alteration of physiological splicing of exon 28. Two independent transfections are shown. **(D)** Schematic representation of minigene products. Both transcripts lacked exon 29 which is alternatively spliced into embryonic tissues.

To analyze the effects of c.3526-2A>G we used a minigene assay. Genomic fragments comprising exons 26 to 30 were amplified from Proband II.1 (Figure 3A). After transfection, RNA was analyzed by reverse-transcription PCR. The PCR product belonging to the plasmid containing the c.3526-2A>G had a lower molecular weight than that obtained from the plasmid with the WT sequence (Figure 3B). Sequencing revealed loss of exon 28 in the lower band and absence of exon 29 in both transcripts (Figures 3C, D). Thus, the mutation disrupts the acceptor splice site of intron 27 and causes the skipping of exon 28. Exon 28 is in-frame and codes for 28 amino acids that correspond to the transmembrane segment S3 of domain IV that is part of the voltage-sensing domain. The skipping of exon 28, though resulting in an in-frame deletion in the transcript, could have functional consequences: (i) the loss of a transmembrane segment which can disrupt proper protein folding and trafficking to the membrane; (ii) the alteration of the region around DIV S3-S4 linker that regulates the voltage dependence of activation through alternative splicing of exon 29 (11). Furthermore, DIV-S3 contains negatively charged D1186 (D3) and D1196 (D4) which interact with the positively charged arginines of DIV-S4 throughout the conformational changes that occur during the transition from the resting state to the activated state of Ca_v_1.1 (5). Thus, the lack of exon 28 could affect both the embryonic and the adult Ca_v_1.1 isoforms.

In our *in vitro* model, exon 29 is skipped both in the mutant and in the WT transcripts. We used HEK cells that are of embryonic origin, and exon 29 is physiologically skipped in embryonic tissues (6).

### 2.4 Patient perspective

The patients' mother relayed challenges to the girls being able to participate in daily domestic activities and societal activities that are a significant aspect of their self-identity within the community. She was particularly fearful the disease would affect their ability to care for their families in the future.

## 3 Discussion

The clinical presentation of congenital myopathy phenotype compounded by very early onset episodic weakness and electrical myotonia is distinct from previous phenotypic descriptions reported with *CACNA1S* mutations. Atypical features included episodic weakness prior to 1 year of age, short duration of episodes, normal short and long exercise testing, and exacerbation by cold exposure. Genetic testing revealed two mutations in the *CACNA1S* gene, which encode the α1 pore-forming subunit of the DHPR, a voltage-gated calcium channel responsible for ECC along with RyR1.

The diagnosis of a congenital myopathy in our probands rested on the clinical picture, considering normal CPK levels and the absence of muscle pathology evaluation. This was based on the presence of weakness and hypotonia noted shortly after birth, coupled with the static nature of the weakness, and a genotype associated with congenital myopathy. Previously described *CACNA1S*-related congenital myopathy patients all had normal CPK levels, and non-specific muscle biopsy findings including fiber size disproportion and a non-specific myopathic appearance (Supplementary Table 1). However, a muscle pathology description would have had a positive impact on the interpretation of our observed phenotype and added further insight into the phenotypic expression of *CACNA1S* related mutations.

Historically, congenital myopathies have been classified on the basis of major morphological features seen on muscle biopsy (12). As more genes are identified, newer classification systems will likely be necessary taking into consideration the underlying genetic etiology.

*In vitro* minigene splicing assay demonstrated that the c.3526-2A>G mutation disrupts the acceptor splice site of intron 27, leading to skipping of exon 28, an in-frame exon that codes for S3 in the fourth domain. The fourth voltage-sensor domain comprises a functionally critical intramolecular interaction between oppositely charged residues of the S3 and S4 transmembrane helices; this interaction is required for proper voltage sensitivity of Ca_v_1.1 (13). It has also been demonstrated that the structure and length of the fourth S3–S4 linker is critical in regulating the voltage sensitivity of the fourth voltage-sensor domain (5). Moreover, alternative splicing of exon 29 controls the length of the DIV S3–S4 linker and regulates voltage dependence of activation (14). Thus, this mutation is expected to affect channel function by disrupting the integrity of the interaction between the S3 segment and the voltage sensor S4, and by altering the S3–S4 linker region and thus affect voltage dependence of activation (15).

In *CACNA1S*-related congenital myopathy cases, truncating mutations leading to a premature termination codon, or missense mutations leading to decreased protein stability all lead to absence or reduction of protein amounts (10). Although the deletion caused by the mutation in our patients does not alter the reading frame, the lack of an entire transmembrane segment could prevent the correct folding of the protein and its trafficking to the membrane, likely leading to reduction in Ca_v_1.1 level. The decreased content of Ca_v_1.1 also impairs ECC (10) thus, changes in excitation-contraction coupling are likely to contribute to the weakness and myopathy features encountered (4, 11). The *in vitro* minigene splicing assay is a powerful tool for predicting consequences in pre-mRNA maturation, however a functional assay that directly evaluates the impact of a variant on channel function is the only tool capable of revealing whether this variant is pathogenic. Different mechanisms of genetic regulation, such as activation of alternative splice sites, could intervene and change the expected effect.

While most mutations thus far identified in the *CACNA1S* as causes of HypoPP have been in the highly conserved S4 segments (16), novel mutations located in new regions of the *CACNA1S* are increasingly being identified (15). Two of these mutations fall into the third domain near p.Cys944Tyr: p.His916Gln, located in the DIII S4-S5 linker, and p.Glu989Lys, located in the DIII S5–S6 linker (17, 18). The p.Cys944Tyr variant can contribute to the episodic weakness phenotype in the two sisters. So far, only one patient has been described as having congenital myopathy plus episodes of periodic paralysis starting at the age of 5 years which aggravated the congenital phenotype (10, 19).

Our patient's phenotype included electrical myotonic discharges without clinical myotonia. This is not a feature characteristic of the hypokalemic periodic paralysis associated with mutations in *CACNA1S*. However, our patients' phenotype is mainly that of a congenital myopathy, compounded by superimposed episodic weakness. As such, myotonic discharges in the absence of clinical myotonia have been reported in several genetic muscle disorders and congenital myopathies, involving genes not typically correlated with myotonia (20, 21).

To date, 13 patients from 9 unrelated families have been described with *CACNA1S*-related recessive congenital myopathy (Supplementary Table 1) (10, 19, 22–26). The phenotypes described are characterized by antenatal or neonatal onset of symptoms (9/9 families), delayed motor development (7/7 families), respiratory involvement (6/8 families), feeding difficulties (8/8 families), and facial involvement often characterized by ophthalmoplegia (7/7 families). Periodic weakness was reported in 1/13 patients. All had normal CK levels. The alveolar aspect of the intermyofibrillar network seen on muscle biopsy was described as a possible pathophysiological hallmark for both recessive and dominant *CACNA1S*-related congenital myopathy. However, in recessive patients this feature has only been described in 2/8 families. Although this is a small series of patients, there is considerable variability in the severity of the phenotype and no genotype-phenotype correlation emerges. The phenotype of our patients ranks among the less severe on the phenotypic spectrum.

This report expands our knowledge on *CACNA1S*-related congenital myopathy, an entity described only a few years ago, and supports the notion that various phenotypes represent a continuum on the clinical spectrum associated with these mutations.

## Data availability statement

The datasets presented in this article are not readily available because of ethical and privacy restrictions. Requests to access the datasets should be directed to the corresponding author.

## Ethics statement

The studies involving humans were approved by North Jordan Institutional Review Board. The studies were conducted in accordance with the local legislation and institutional requirements. Written informed consent for participation in this study was provided by the participants' legal guardians/next of kin. Written informed consent was obtained from the minor(s)' legal guardian/next of kin for the publication of any potentially identifiable images or data included in this article.

## Author contributions

SA: Conceptualization, Data curation, Investigation, Methodology, Writing – original draft, Writing – review & editing. LR: Investigation, Writing – review & editing. MS: Resources, Investigation, Writing – review & editing. FB: Investigation, Resources, Writing – review & editing. SL: Investigation, Writing – review & editing. GC: Resources, Methodology, Writing – review & editing. GM: Conceptualization, Investigation, Methodology, Project administration, Writing – review & editing. SP: Data curation, Methodology, Resources, Writing – original draft, Writing – review & editing.
