# Supplementary material for: Case report: Dihydropyridine receptor (CACNA1S) congenital myopathy, a novel phenotype with early onset periodic paralysis
Source: Front Neurol. 2024 Feb 15;15:1359479. doi: 10.3389/fneur.2024.1359479 (PMC10902085; doi:10.3389/fneur.2024.1359479)
Supplement: Supplementary file 1 [file Data_Sheet_1.docx]

**Supplemetary Table 1**

Previously described cases of *CACNA1S*-related congenital myopathy

|  | **Age at description** | **Genetics** | **Onset** | **Muscle weakness** | **Ophthalmoplegia, ptosis, weak face** | **Respiratory involvement** | **Feeding** | **Biopsy** | **MRI** | **Follow up** |
| --- | --- | --- | --- | --- | --- | --- | --- | --- | --- | --- |
| **Hunter, 2015** 1 patient | 3 months | c.3795G>T, p.Gln1265His; c.4947delA, p.Gln1649Glnfs*72 | Neonatal | Severe generalized congenital weakness | Ophthalmoplegia, high arched palate | Labored, paradoxical | No sucking or swallowing Gastrostomy | Fiber size variability, whorled fibers | Brain; normal | n/a |
| **Schartner, 2017** 1 patient | 60 years | c.1189_1190del, p.Ser397Profs*3; c.4967del, p.Leu1656Argfs*67 | Antenatal, neonatal | Axial, proximal, distal weakness | Ophthalmoplegia, high arched palate | Normal | Mild dysphagia | Fiber size variability, centralized nuclei | Muscle atrophy | Severe axial weakness |
| **Schartner, 2017** 1 patient | 16 years | c.4453C>T, p.Gln1485*; c.4967del, p.Leu1656Argfs*67 | Antenatal, neonatal | Limb girdle, axial weakness | Ophthalmoplegia, high arched palate | Mild weakness | Mild dysphagia | Fiber size variability, centralized nuclei | Fatty muscle, anterior lower limb replacement | Continued weakness |
| **Schartner, 2017** 2 patients | 15 years  7 years | c.825C>A, p.Phe275Leu; c.2371delC, p.Leu791Cysfs*37 | Neonatal | Stable diffuse weakness | High arched palate | Normal | Gastrostomy in 1 patient | n/a | Mild vastus lateralis changes | Mild stable weakness |
| **Schartner, 2017 Matthews, 2019** 1 patient | 10 years | c.298G>T, p.Glu100Lys; c.3795G>T, p.Gln1265Hisfs*57 | Antenatal, neonatal | Generalized; periodic weakness and loss of speech | High arched palate, ophthalmoplegia, ptosis | Severe weakness | Intermittent feeding difficulties | Fiber size variability, endomysial connective tissue | Marked atrophy of upper leg muscles, fatty muscles | Improvement after acetazolamide |

**Supplementary Table 1 continued**

Previously described cases of *CACNA1S*-related congenital myopathy

|  | **Age at description** | **Genetics** | **Onset** | **Muscle weakness** | **Ophthalmoplegia, ptosis, weak face** | **Respiratory involvement** | **Feeding** | **Biopsy** | **MRI** | **Follow up** |
| --- | --- | --- | --- | --- | --- | --- | --- | --- | --- | --- |
| **Yis, 2019**  3 patients | 1 at 5 years 2 deceased | c.2366G>A, p.Arg789His homozygous | Antenatal, neonatal | Muscle weakness | Ophthalmoplegia | Respiratory weakness, tracheostomy | Absent suck in all siblings Gastostomy in 1 sibling | Mild dystrophic changes, centralized nuclei, fibrosis | Brain; mild ventricular enlargement | Continued severe weakness |
| **Ravenscroft, 2020** 2 patients | 1 neonatal 1 fetal death | c.665T>A, p.Met222Lys; c.2365C>T, p.Arg789Cys | Antenatal | Fetal akinesia | n/a | n/a | n/a | No atrophy, no myofibrillar disorganization | n/a | n/a |
| **François-Heude, 2021** 1 patient | 1 year | c.2618del, p.Leu873Argfs*21; c.5104C>T, p.Arg1702* | Neonatal | Muscle weakness | Amimia | Respiratory weakness, CPAP | Gastrostomy | Centronuclear myopathy | Normal muscle MRI | Progressive improvement |
| **Juntas Morales, 2021** 1 patient | 30 years | c.2970G>A, p.Trp990*; c.5104C>T, p.Arg1702* | Neonatal | Mild axial and proximal weakness | n/a | Respiratory insufficiency | Feeding difficulty | Nonspecific myopathic pattern | Upper and lower limb atrophy | Progressive improvement |
| **Present study** 2 patients | 12 years 4 years | c.2831G>A, p.Cys944Tyr; c.3526-2A>G, p.Gly1176_Asp1203 | Neonatal | Mild proximal weakness | Mild facial weakness | Normal | Normal | n/a | Bilateral glutei maximus fat replacement, atrophy | Continued weakness aggravated by cold exposure |

Abbreviations: n.a. (not available)
